# Editorial: New advances in dietary fibers and their role in metabolic, digestive, and immune health

**DOI:** 10.3389/fnut.2024.1404346

**Published:** 2024-04-05

**Authors:** Junrui Cheng, Aylin Sahin, Changling Hu, Renee Korczak, Jing Zhou

**Affiliations:** ^1^Ingredion Incorporated, Bridgewater, NJ, United States; ^2^School of Food and Nutritional Sciences, College of Science, Engineering and Food Science, University College Cork, Cork, Ireland; ^3^Laboratory for Functional Food and Human Health, Center for Excellence in Post-Harvest Technologies, North Carolina Agricultural and Technical State University, Kannapolis, NC, United States; ^4^Department of Food Science and Nutrition, University of Minnesota Twin Cities, St. Paul, MN, United States

**Keywords:** dietary fiber, metabolic health, immune health, digestive health, gut health

Global health authorities including FDA, EFSA, Health Canada, etc. have defined dietary fiber as non-digestible carbohydrates that either naturally occur in foods of plant origin, or isolated or synthetic carbohydrates that have demonstrated physiological effects that are beneficial to human health (1). Differing by solubility, fermentability, and their structure, dietary fibers function through diverse mechanisms and pathways, and benefit human health both directly and/or indirectly. The gut microbiome plays a critical role in modulating the metabolic, digestive, and immune health of the host and, when disturbed, may lead to the development of diseases (2, 3). Fermentable dietary fibers provide the main source of energy for the colonocytes and gut microbes (2–4). Microbial fermentation of dietary fibers generates metabolites such as short-chain fatty acids (SCFAs), branched-chain amino acids, and neuro-active chemical substances, which act as paracrine or endocrine signaling molecules in initiating physiological responses (4). Despite a considerable number of studies on dietary fibers and their role in human health, the individual variation on the gut microbiome has introduced complexity when drawing accurate conclusions.

Therefore, new interventional studies or mechanistic investigations on dietary fibers and metabolic, digestive, and immune health will advance our knowledge of the function of dietary fibers in these areas. An augmenting pool of research will also enable health professionals to provide dietary recommendations based on health needs. With this, the aim of this Research Topic is to collect papers suitable to improve our knowledge and understanding on dietary fibers and their role in impacting metabolic, digestive, and immune health.

The diverse landscape of research on dietary fibers has seen remarkable advancements, as evidenced by the compelling array of publications in this Research Topic. In this Research Topic there are ten papers covering the above-mentioned aspects. From elucidating the impacts of low carbohydrate diets to exploring the immunomodulatory effects of specific dietary fibers, each study contributes to our understanding of the intricate interplay between nutrition and gastrointestinal wellbeing.

One noteworthy trend highlighted in these publications is the growing recognition of the pivotal role played by dietary fibers and microbial fermentation products, such as short-chain fatty acids (SCFAs), in maintaining gut homeostasis and fostering overall health (Ashique et al., Cheng and Zhou, Bacha et al., Li et al., Sheng et al., Singh and Bhardwaj, Qi et al.). In addition, there is a growing emphasis on understanding the intricate crosstalk between the gut and other organs (Cheng and Zhou, Li et al., Sheng et al., Singh and Bhardwaj, Qi et al.). This emerging focus underscores the profound impact of gut metabolites on systemic physiology and disease pathogenesis. Studies investigating the immunomodulatory effects of inulin and its intestinal metabolites, for instance, shed light on the intricate signaling pathways through which gut-derived compounds exert far-reaching effects on immune function and inflammatory processes beyond the confines of the gastrointestinal tract (Sheng et al.). In addition to inulin, studies examining the effects of other prebiotics including β-glucans, High-Amylose Maize Starch Butyrate (HAMSB), psyllium husk fiber, and a variety of viscous soluble dietary fibers, as well as synbiotics underscore their potential as valuable tools for modulating gut microbiota composition and bolstering immune function (Cheng and Zhou, Bacha et al., Lu K. et al., Singh and Bhardwaj). Furthermore, investigations into the mechanisms of butyrate, particularly its therapeutic potential in addressing colorectal disturbances, offer promising avenues for clinical intervention (Cheng and Zhou, Bacha et al., Sheng et al., Singh and Bhardwaj).

However, amidst these strides, certain studies also shed light on areas warranting further exploration and clarification. For instance, the findings regarding pea hull fiber supplementation in individuals undergoing hemodialysis emphasize the need for nuanced approaches tailored to specific populations (Fatani et al.), highlighting the complexity inherent in assessing dietary interventions across diverse health conditions. Furthermore, as highlighted by the investigation into the effect of viscous soluble dietary fiber on glucose and lipid metabolism in patients with type 2 diabetes mellitus, personalized nutrition approaches can offer tailored solutions to address specific metabolic imbalances and optimize health outcomes for individuals with distinct physiological profiles (Lu K. et al.). Another evidence-based study emphasized the role of synbiotics in supporting the management of ulcerative colitis (Ashique et al.). By integrating advanced omics technologies with comprehensive lifestyle assessments, personalized nutrition strategies can provide nuanced insights into the complex interactions between diet, genetics, and microbiome composition, paving the way for precision health interventions tailored to individual needs. Meanwhile, the study using NHANES data highlighted a benefit of consuming more fibers in combating inflammation and improving immune health (Qi et al.), indicating that the holistic impact of dietary choices on overall wellbeing cannot be overstated.

As we navigate this complex terrain, it is imperative to adopt an integrative approach that encompasses not only the direct effects of dietary components on gut health but also their broader implications for immune and metabolic health (Lu G. et al.). Furthermore, ongoing efforts to elucidate the underlying mechanisms driving these interactions will be essential for informing targeted dietary interventions and optimizing health outcomes across diverse populations.

In conclusion, the breadth and depth of research showcased in this Research Topic underscore the dynamic nature of the field of dietary fiber research. By synthesizing insights from these diverse studies, we move closer toward unraveling the intricacies of the gut microbiome and harnessing its therapeutic potential to promote optimal health and wellbeing.

## Author contributions

JC: Writing – original draft. AS: Writing – review & editing. CH: Writing – review & editing. RK: Writing – review & editing. JZ: Writing – review & editing.

## References

[1] Stephen AM Champ MM Cloran SJ Fleith M van Lieshout L Mejborn H. Dietary fibre in Europe: current state of knowledge on definitions, sources, recommendations, intakes and relationships to health. Nutr Res Rev. (2017) 30:149–90. 10.1017/S095442241700004X 28676135

[2] Zheng D Liwinski T Elinav E. Interaction between microbiota and immunity in health and disease. Cell Res. (2020) 30:492–506. 10.1038/s41422-020-0332-7 32433595 PMC7264227

[3] Vijay A Valdes AM. Role of the gut microbiome in chronic diseases: a narrative review. Eur J Clin Nutr. (2022) 76:489–501. 10.1038/s41430-021-00991-6 34584224 PMC8477631

[4] Fu J Zheng Y Gao Y Xu W. Dietary fiber intake and gut microbiota in human health. Microorganisms. (2022) 10:2507. 10.3390/microorganisms10122507 36557760 PMC9787832

